# Incomplete Excision Rate for Lentigo Maligna and Associated Risk Factors

**DOI:** 10.2340/actadv.v104.40535

**Published:** 2024-10-03

**Authors:** Maja MODIN, Helena SVENSSON, Ylva Bergsten WANDERS, Noora NEITTAANMÄKI, Jan SIAROV, John PAOLI

**Affiliations:** 1 Department of Dermatology and Venereology, Institute of Clinical Sciences, Sahlgrenska Academy, University of Gothenburg; 2 Region Västra Götaland, Sahlgrenska University Hospital, Department of Dermatology and Venereology, Gothenburg; 3 Department of Laboratory Medicine, Institute of Biomedicine, Sahlgrenska Academy, University of Gothenburg; 4 Region Västra Götaland, Sahlgrenska University Hospital, Department of Clinical Pathology, Gothenburg, Sweden

**Keywords:** dermatological surgical procedures, incomplete excision, lentigo maligna, margins of excision, melanoma, surgery

## Abstract

Standard treatment for lentigo maligna (LM) is surgical excision, yet insights into the frequency of and risk factors for incomplete excisions remain limited. The primary objectives were to assess the incomplete excision rate (IER) in primary LM and to explore potential risk factors for incomplete excisions. A retrospective analysis was conducted encompassing consecutive histopathologically confirmed LMs from 2014–2020. Descriptive statistics were used for LM characteristics and IER, while uni- and multivariate analyses were used for calculating risk factors. The study included 395 LMs with an IER of 16.7% (*n* = 66). Risk factors for higher incomplete excision rates included: head and neck lesions (*p* = 0.0014), clinical excision margins < 5 mm (*p* = 0.040), and utilization of preoperative partial biopsies (*p* = 0.023). Plastic surgeons had higher IERs than dermatologists (*p* = 0.036). Lesion diameter (*p* = 0.20) and surgeon experience (*p* = 0.20) showed no associations with incomplete excisions, yet LMs with a diameter ≥ 20 mm exhibited higher incomplete excision rates (23.2%) compared witho those < 10 mm (12.9%). LMs should be excised with at least 5-mm clinical margins, especially in the head and neck area. LMs ≥ 20 mm may be more surgically challenging. High-er incomplete excision rates associated with the use of preoperative biopsies and/or plastic surgeons may reflect challenging anatomical locations, larger lesion diameter, and/or ill-defined borders.

## SIGNIFICANCE

Surgical excision is the most commonly used treatment for lentigo maligna, but the frequency of incomplete excisions and associated risk factors have not been sufficiently analysed. This report shows that 1 out of 6 lentigo malignas are incompletely excised. Location in the head and neck area, clinical excision margins < 5 mm, utilization of preoperative partial biopsies and surgeon specialty (plastic surgeon) were associated with higher incomplete excision rates. This knowledge may reduce incomplete excision rates for lentigo maligna in the future leading to less undue suffering for patients, less risk of functional and cosmetic impairment, and lower expenses.

Lentigo maligna (LM), a melanoma in situ in chronically sun-damaged skin, originates de novo in a lentiginous growth pattern, where atypical melanocytes align along the epidermal basal layer. With slow progression and horizontal growth, it can persist for decades without transforming into invasive lentigo maligna melanoma (1). If transformation occurs, the lesion may become more aggressive and resemble other invasive melanomas (2, 3).

The annual report of new cases of LM in Sweden has doubled during the last decade to about 1,000 cases per year. Consequently, LMs account for approximately a quarter of all cases of melanoma *in situ* in Sweden (4). This increase may reflect improved diagnostic capabilities, sun exposure habits, and the growing senior population in Sweden.

Incomplete excisions result in further surgery inflicting undue patient suffering, increase the risk of functional and cosmetic impairments, and result in additional costs for the healthcare system. In medical care, LM generates clinical, surgical, and histopathological challenges. The clinical diagnostic accuracy is obscured due to the resemblance to other premalignant and benign lesions (5, 6). Nevertheless, recently described dermoscopic findings may facilitate the diagnosis (2, 7–9). Reflectance confocal microscopy can facilitate preoperative diagnosis and demarcation of LM (10), but this technique is expensive, labour-intensive, and has limited access. The frequent occurrence in the head and neck area, where excess skin is limited, adds complexity to the surgical removal. Furthermore, the radial diffuse growth of LM and the presence of surrounding actinic melanocytic hyperplasia in sun-damaged skin aggravate histopathological diagnosis and clearance assessment (11, 12).

The most common treatment for LM is surgical excision, i.e., excision with a fixed safety margin without intraoperative margin control. The aim is to achieve complete clearance, i.e., histopathologically confirmed clear margins. However, evidence regarding optimal management of LM is deficient and randomized controlled trials for assessment of appropriate clinical excision margins for LM are lacking. Present international guide-lines generally recommend clinical margins of 5–10 mm (5, 13–15).

The sufficiency of 5-mm clinical margins to achieve complete histopathological clearance has been questioned in previous studies (16–19). However, these studies based their conclusions on outcomes following more complex surgical methods (e.g., Mohs micrographic surgery and staged excision). Moreover, they included more advanced LMs and/or other types of melanomas compromising external validity. Prior retrospective studies on the incomplete excision rate (IER) for LM using surgical excision have reported great variation ranging from 8% to 29% (20–22). A recent prospective study employing staged excisions on 438 LMs revealed the need for wider clinical margins to ensure complete excision of lesions with a larger diameter (23). Beyond this, risk factors associated with higher IERs for LM are not well known.

The aim of this study was to analyse the IER in the initial treatment of histopathologically verified LM using surgical excision and to evaluate potential clinical risk factors associated with incomplete excisions.

## MATERIALS AND METHODS

We conducted a retrospective study on primary, histopathologically confirmed LMs removed using surgical excision, i.e. without intraoperative margin control, between January 1, 2014, and December 31, 2020. These cases were identified through the pathology register at the Department of Pathology at Sahlgrenska University Hospital in Gothenburg, Sweden utilizing the SNOMED code M87422. Previously untreated LMs excised with the intention to achieve complete excision were included. The only exclusion criterion was lack of data regarding margin control (i.e., whether the excision was complete or incomplete) as indicated in the pathology report. Due to the nature of this cross-sectional, retrospective study, no clinical follow-up assessments were conducted. This study was authorized by the Regional Ethical Review Board in Gothenburg, Sweden and the Swedish Ethical Review Authority.

Incomplete excisions were defined as cases in which tumour cells were visible at the histopathological specimen border or uncertain margin control was observed in the histopathological report. Given the retrospective study design, no pre-established criteria for defining positive/negative margins were used. Generally, cellular atypia and architectural growth are considered and positive margins show higher melanocytic density compared with chronically sun-damaged skin (24). Histopathological diagnosis and margin control were based on routine “bread-loaf” cross-sections with haematoxylin-eosin staining and, in challenging cases, with immunohistochemistry (IHC) (25, 26). In order to investigate potential clinical risk factors associated with incomplete excision, demographic, tumour-related, surgery-related, and surgeon-related data (**Table I**) were collected from pathology reports and electronic patient records. To ensure consistent data collection, a standard protocol was created. For example, clinical excision margins documented as “4–5 mm” were categorized as 4 mm and lesion diameter was defined as the maximum diameter stated in any of the records.

**Table I:** Collected data from pathology reports and electronic patient records

Clinical/surgical category	Clinical/surgical parameter	Clinical/surgical sub-parameter	Unit
**Demographic data**	Sex		Female
			Male
	Age		Years
**Tumo**u**r-related data**	Anatomic location	Head and neck	Cheek
			Forehead
			Nose
			Periorbital
			Neck
			Ear
			Scalp
			Perioral
		Trunk	
		Upper extremities	Arm
			Hand
		Lower extremities	
	Lesion diameter		mm
**Surgery-related data**	Excision margin	Clinical	mm
		Histopathological	mm
	Preoperative biopsy		Yes/No
**Surgeon-related data**	Surgeon specialty		Dermatologist
			Plastic surgeon
			Otorhinolaryngologist
			General surgeon
			General practitioner
			Other specialties
	Surgeon experience level		Specialist
			Resident

### Statistical analysis

To outline the LM characteristics, descriptive statistics were utilized. Other statistical analyses were conducted using R version 3.5.3 (R Foundation for Statistical Computing, Vienna, Austria). Fisher’s exact test was applied to compare proportions between 2 independent groups, while Wilcoxon’s rank-sum test was utilized for two-sample tests in univariate analyses. To adjust for possible confounders, multiple logistic regression was applied. All statistical tests were two-sided and *p* < 0.05 was considered statistically significant.

## RESULTS

A total of 395 lesions in 380 patients (51.8% female) were included in the study. The median patient age was 75 years (range 42–96 years) with no significant difference between the sexes (*p* = 0.24). In this material, 158 lesions (40.0%) were assessed with one or more IHC stains: Melan-A (*n* = 95, 24.1%), SOX-10 (*n* = 81, 20.5%), S100 (*n* = 46, 11.6%), HMB45 (*n* = 14, 3.5%), and others (*n* = 6, 1.5%). In total, 66 lesions were incompletely excised, resulting in an IER of 16.7%. The proportions of incompletely excised lesions associated with possible risk factors are presented in **Table II**.

**Table II:** Clinical and surgical categories and their univariate association with incomplete excisions of lentigo maligna

Clinical/surgical category	Lentigo maligna *n* (%)	Incomplete excisions *n* (%)	*p*-value
Anatomic location			
Head and neck	324 (82.0)	61 (18.8)	**0.0014**
Trunk and extremities	71 (18.0)	5 (7.0)	
Clinical excision margin			
< 5 mm	43 (10.8)	11 (25.6)	**0.040**
5 mm	300 (75.9)	39 (13.0)	
6–9 mm	5 (1.3)	0 (0.0)	
10 mm	5 (1.3)	1 (20.0)	
Unknown	42 (10.6)	15 (35.7)	
Surgeon specialty			
Dermatologist	174 (44.0)	19 (10.9)	**0.036**
Plastic surgeon	173 (43.8)	37 (21.4)	
Otorhinolaryngologist	29 (7.3)	7 (24.1)	
General surgeon	12 (3.0)	1 (8.3)	
General practitioner	3 (<1)	1 33.3)	
Other specialty	3 (<1)	1 (33.3)	
Unknown	1 (<1)	0 (0.0)	
Preoperative biopsy			
Yes	255 (64.6)	51 (20.0)	**0.023**
No	140 (35.4)	15 (10.7)	
Lesion diameter			
< 10 mm	116 (29.3)	15 (12.9)	0.20
10–19 mm	191 (48.4)	30 (15.7)	
≥ 20 mm	69 (17.5)	16 (23.2)	
Unknown	19 (4.8)	5 (26.3)	
Experience level			
Specialist	322 (81.5)	58 (18.0)	0.20
Resident	66 (16.7)	7 (10.6)	
Unknown	7 (1.8)	1 (14.3)	

### Tumour-related factors

The majority of the lesions (*n* = 324, 82.0%) were situated in the head and neck area, with the most prevalent locations being the cheek (38.2%), followed by the forehead (12.2%), and the nose (10.9%). Among lesions in other anatomical areas (*n* = 71, 18.0%), the trunk and upper extremities were the most common (8.4% and 7.3%, respectively). No significant difference in lesion diameter was observed between the head and neck area and other anatomical sites (*p* = 0.99). However, a significant difference in lesion location was noted between men and women (*p* < 0.001), with men exhibiting a higher prevalence of lesions on the scalp (8.3% vs 0%) and ear (7.3% vs 1.5%). In contrast, lesions on the extremities were more common in women (12.8% vs 5.7%) (*p* = 0.0005). IERs were also significantly higher in women (20.7% vs 12.5%) (*p* = 0.031).

Among the incompletely excised lesions, 92.4% (*n* = 61) were located in the head and neck area and 7.6% (*n* = 5) on the trunk or the extremities (1 on the dorsum of the hand). There was a significantly higher IER in the head and neck area compared with other anatomic locations (*p* = 0.0014). The location with the highest IER was the periorbital area (40.0%, *n* = 10). **Table III** demonstrates the specific anatomical locations for all lesions and the respective IERs.

**Table III:** Frequency and proportion of incompletely excised lentigo maligna according to specific anatomical locations

Anatomic location	Lentigo maligna *n* (%)	Incomplete excisions *n* (%)	Incomplete excisions (%) 95% CI
**Head and neck**	**324 (82.0)**	**61 (18.8)**	**14.7–23.5**
Cheek	151 (38.2)	25 (16.6)	11.0–23.5
Forehead	48 (12.2)	9 (18.8)	8.9–32.6
Nose	43 (10.9)	9 (20.9)	10.0–36.0
Periorbital	25 (6.3)	10 (40.0)	21.1–61.3
Neck	18 (4.6)	3 (16.7)	3.6–41.4
Ear	17 (4.3)	3 (17.6)	3.8–43.4
Scalp	16 (4.1)	1 (6.3)	0.2–30.2
Perioral	6 (1.5)	1 (16.7)	0.4–64.1
**Trunk**	**33 (8.4)**	**1 (3.0)**	**0.1–15.8**
**Upper extremities**	**29 (7.3)**	**3 (10.3)**	**2.2–27.4**
Arm	28 (7.1)	2 (7.1)	0.9–23.5
Hand	1 (0.3)	1 (100.0)	2.5–100.0
**Lower extremities**	**9 (2.3)**	**1 (11.1)**	**0.3–48.2**

CI: confidence interval.

The median lesion diameter was 11 mm (range 2–70 mm). The median diameter of incompletely excised lesions (12 mm) was not significantly different from that of completely excised lesions (11 mm) (*p* = 0.21). LMs < 10 mm in diameter were incompletely excised less frequently (12.9%) than LMs ≥ 20 mm (23.2%), but no statistically significant differences were observed (*p* = 0.20).

### Surgery-related factors

The clinical excision margin was known in 89.4% of all cases. Most lesions (85%, *n *= 300) were excised with a 5-mm clinical margin. The mean clinical margin was 4.8 mm (range 1–10 mm). In 2.5% of cases (*n* = 10), clinical margins > 5 mm were applied, while in 10.9% (*n* = 43), margins < 5 mm were used.

The mean clinical margin in incompletely excised lesions was 4.6 mm (95% CI 4.3–5.0) compared with 4.8 mm in the completely excised lesions (95% CI 4.7–4.9) (*p* = 0.042). When clinical margins < 5 mm were used, the IER was significantly higher than when exactly 5-mm clinical margins were used (25.6% and 13.0%, respectively) (*p* = 0.036). The vast majority of LMs excised with clinical margins < 5 mm were located in the head and neck area (93.0%).

Data on histopathological margins were available for 66.6% (*n* = 219) of the completely excised lesions. Among these, 68.5% (*n* = 150) had a histopathological margin ≥ 2 mm, and 2.7% (*n* = 6) were reported to have “adequate margins”. In 24.7% of cases (*n* = 54), histopathological margins < 2 mm were observed and another 4.1% (*n* = 9) were labelled as having “narrow margins”.

Overall, preoperative biopsies were performed in 64.6% of all cases (*n* = 255). Lesion diameter was significantly larger in cases in which preoperative biopsies were conducted with a median size of 12 mm compared with 10 mm in cases without preoperative biopsies (*p* = 0.008). No significant differences in clinical excision margins were found for lesions diagnosed with or without a preoperative biopsy (*p* = 0.17). Of the lesions in which preoperative biopsies were performed, 51 lesions (20.0%) were incompletely excised compared with 15 lesions (10.7%) in the group in which no preoperative biopsy was performed (*p* = 0.023).

### Surgeon-related factors

The majority of excisions (87.8%) were conducted by dermatologists (*n* = 174) or plastic surgeons (*n* = 173). Plastic surgeons excised a significantly higher proportion of lesions in the head and neck area (*n* = 167, 96.5%) compared with dermatologists (*n* = 122, 70.1%) (*p* < 0.001). Median lesion diameter differed significantly, with dermatologists excising lesions with a median size of 10 mm (range 2–30 mm) and plastic surgeons excising LMs with a median size of 15 mm (range 2–70 mm), (*p* < 0.001). Preoperative biopsies were more common in lesions excised by plastic surgeons (*n* = 149, 86.1%) compared with those excised by dermatologists (*n* = 81, 46.4%) (*p* < 0.001). The IER was lower in excisions performed by dermatologists compared with plastic surgeons (*p* = 0.009). A difference could be seen in IERs when further dividing the surgeon specialties into dermatologists in public healthcare (*n* = 140, IER 8.6%), dermatologists in private healthcare (*n* = 34, IER 21.4%), and plastic surgeons (*n* = 173, IER 20.6%) (*p* = 0.005).

Data on clinical excision margins, known in 89.1% (*n* = 352) of cases, exhibited significant differences among surgeon specialties (*p* < 0.001). Dermatologists and plastic surgeons, who performed the majority of surgeries, used mean clinical excision margins of 4.8 mm (*n* = 147, 95% CI 4.7–5.0) and 4.9 mm (*n* = 170, 95% CI 4.7–5.1), respectively. General surgeons employed a mean clinical margin of 5 mm (*n* = 11, 95% CI 5.0–5.0), while otorhinolaryngologists used a lower mean clinical margin of 3.8 mm (*n* = 21, 95% CI 3.1–4.5). Data were insufficient to analyse mean clinical margins used in the remaining excisions conducted by other specialties (*n* = 3).

Specialists excised 81.5% (*n* = 322) and residents excised the remaining 16.7% (*n* = 66) of all LMs for which data on surgeon experience was available. No significant difference could be seen in IERs between specialists and residents (*p* = 0.20). Specialists performed a higher proportion of excisions in the head and neck area (85.1%, *n* = 274) compared with residents (71.2%, *n* = 47) (*p* = 0.012) and preoperative biopsies were performed more often in the cases excised by specialists compared with those excised by residents (67.7%, *n* = 218, and 51.5%, *n* = 34, respectively, *p* = 0.016). No significant difference regarding lesion diameter could be seen between specialists and residents (*p* = 0.73) or in clinical excision margins between specialists and residents (*p* = 0.14).

### Multivariate analysis

A multivariate analysis was performed including the following statistically significant univariate variables: anatomic location (head and neck vs other locations), surgeon specialty (dermatologist vs plastic surgeon), clinical excision margin (5 mm versus < 5 mm) and preoperative biopsy (no vs yes). Only surgeon specialty was confirmed to be statistically significant, with plastic surgeons showing a higher IER compared with dermatologists (*p* = 0.0080) (**Table IV**).

**Table IV:** Multivariate analysis of the statistically significantly variables associated with incomplete excision of lentigo maligna upon univariate analysis

Clinical/surgical categories	*p*-value
Anatomic location (head and neck vs other)	0.58
Clinical excision margin (5 mm vs < 5 mm)	0.11
Surgeon specialty (dermatologist vs plastic surgeon)	**0.008**
Preoperative biopsy (yes vs no)	0.76

## DISCUSSION

In this cohort of 395 LMs undergoing surgical excision, approximately 1 out of 6 were incompletely excised. IERs were reduced to 1 out of 8 when 5-mm clinical margins were used. Anatomic location in the head and neck area, clinical excision margins < 5 mm, surgeon specialty (plastic surgeons vs dermatologists), and the use of preoperative biopsies were associated with higher IERs in the univariate analysis, while lesion diameter and surgeon experience level were not. In the subsequent multivariate analysis, only the surgeon’s specialty (plastic surgery) was confirmed as statistically significant.

Lesions excised in the head and neck area exhibited a higher IER, aligning with previous studies (18, 27, 28). This can be attributed to several plausible factors. The lentiginous growth pattern of LM on sun-damaged skin in the head and neck area may pose challenges in identifying the clinical borders of the lesion accurately. Surgical complexities related to maintaining both functionality and cosmetic outcomes may compromise the feasibility of achieving the minimum 5-mm clinical excision margins in this area. This is supported by the significant proportion of head and neck lesions in our study, which were excised with < 5-mm clinical margins. On the trunk and extremities, tissue-sparing techniques are generally less imperative, leading to relatively straightforward reconstructions in these areas. In contrast, the periorbital region, considered one of the most surgically challenging locations, exhibited the highest IER at 40%.

LMs subjected to preoperative biopsies exhibited a higher IER than those without such biopsies. Notably, lesions undergoing preoperative biopsies were significantly larger and often situated in the head and neck area. The tendency to perform preoperative biopsies, especially in anatomically constrained regions, may stem from a desire to avoid overly aggressive excisions when the diagnosis is uncertain. Preoperative biopsies themselves may not be a direct risk factor for incomplete excision but could serve as a surrogate marker for larger, challenging-to-demarcate LMs in anatomically constrained areas. No prior studies investigating the association between preoperative biopsies and IERs were found.

As mentioned previously, several retrospective, single-centre studies have explored IERs of LM using surgical excision including a combined total of 433 LMs, with reported results ranging from 8.0% to 29.3% (20–22). However, these studies are not directly comparable to the findings presented here. For instance, Osborne and Hutchinson used a standard excision margin of 2 mm for all excisions in their study (22). Akhtar et al. did not specify the clinical margins used (20), and Hou et al. reported only the resulting histopathological margins (21).

Lesion diameter did not significantly differ between completely and incompletely excised cases, consistent with another study on surgical excision for LM (22). However, contrary findings have been reported in studies on melanoma in situ treated with Mohs micrographic surgery or staged excisions (18, 23, 27–30). It is important to note the limited comparability of these studies with ours due to the undisclosed and possibly varying subtypes of melanoma in situ (27–29), use of different surgical techniques like Mohs micrographic surgery (18, 23, 27–30), and different lesion diameters among the included cases (18). Although not statistically significant, our results indicated a trend in which IERs increased with larger lesion diameter. It is worth mentioning that the lesions in our study were relatively small. In our experience, larger LMs often exhibit more ill-defined borders. Furthermore, clinical and dermoscopic features of specific LMs might pose additional risk factors, but these were not explored in this study.

In the majority of cases, 5-mm clinical margins were employed. An elevated IER was observed with margins < 5 mm, aligning with previous studies (17–19, 31). Additionally, studies involving Mohs micrographic surgery or staged excision may exhibit an inclusion bias toward complex cases. Nevertheless, these consistent findings strongly suggest that clinical excision margins < 5 mm significantly increase the risk of higher IERs and should be avoided. Only 10 cases utilized margins > 5 mm, precluding definitive conclusions concerning a potential decrease in IER with larger margins.

Swedish national guidelines advocate complete excision of LMs, aiming at achieving a 2-mm histopathological margin (13). Among the completely excised LMs, 63 had histopathological margins < 2 mm, indicating “narrow margins”. Consequently, 32.7% of lesions (129 cases) would have required additional surgery if national guidelines were strictly followed, raising further concerns about the adequacy of 5-mm clinical excision margins for LM. However, the pros and cons of performing further surgery in elderly patients and/or in anatomically constrained areas despite narrow histopathological margins must be taken into consideration before proceeding.

The multivariate analysis revealed a statistically significant higher IER among plastic surgeons compared with dermatologists. Nevertheless, plastic surgeons handled more cases in the head and neck lesions, cases with preoperative biopsies, and lesions with larger median lesion diameters. IERs also differed between surgeons in public and private practice, but the number of lesions in the last category was too small to analyse further. Notably, previous studies on IERs for LM using surgical excisions did not explore differences in terms of surgeon specialty (20–22).

One of the strengths of this investigation is the inclusion of consecutive cases spanning a 7-year period, providing a relatively large cohort compared with previous studies. Unlike many prior investigations that either excluded LM or focused on melanoma *in situ* without subtyping, our study specifically addresses outcomes for LM. However, it is important to acknowledge the limitation of the retrospective study design without reassessment of histopathological slides or established criteria for evaluation of clear margins, leading to non-standardized and missing data in certain cases. IHC staining, which can improve diagnosis and margin control in excised LM (32), was used in less than half of the included cases. Furthermore, precise diameter measurement may have been underestimated in the few cases in which size was reported based solely on the histopathology report, as tissue may shrink during processing (33). Moreover, exact experience of residents and specialists carrying out the surgery was unknown. A prospective study with well-defined protocols and controlled variables could offer more precise reporting of both clinical and histopathological margins. Additionally, being a single-centre study, our findings are confined to this specific setting, limiting their generalizability to other centres. Lastly, some LMs might not have been registered with the precise SNOMED code and therefore may have been missed in the analysis.

Despite these limitations, our findings emphasize the importance of employing at least 5-mm clinical margins when excising LM, particularly in challenging areas like the head and neck. Surgeons should exercise heightened vigilance when dealing with larger lesions, which often have been biopsied preoperatively. Al-though statistics show that plastic surgeons have higher IERs than dermatologists, the difference may stem from tumour-related factors. These nuances underscore the need for further training of all physicians treating LM in lesion demarcation, histopathological understanding, and tailored surgical approaches using microscopically controlled surgery when available to ensure lower IERs for LM in the future.
